# Anti-thyroid peroxidase antibody and subclinical hypothyroidism in relation to hypertension and thyroid cysts

**DOI:** 10.1371/journal.pone.0240198

**Published:** 2020-10-02

**Authors:** Yuji Shimizu, Shin-Ya Kawashiri, Yuko Noguchi, Yasuhiro Nagata, Takahiro Maeda, Naomi Hayashida

**Affiliations:** 1 Department of Community Medicine, Nagasaki University Graduate School of Biomedical Sciences, Nagasaki, Japan; 2 Department of Cardiovascular Disease Prevention, Osaka Center for Cancer and Cardiovascular Disease Prevention, Osaka, Japan; 3 Center for Comprehensive Community Care Education, Nagasaki University Graduate School of Biomedical Sciences, Nagasaki, Japan; 4 Department of General Medicine, Nagasaki University Graduate School of Biomedical Sciences, Nagasaki, Japan; 5 Division of Strategic Collaborative Research, Atomic Bomb Disease Institute, Nagasaki University, Nagasaki, Japan; National Cerebral and Cardiovascular Center, JAPAN

## Abstract

Hypertension frequently occurs in subclinical hypothyroidism (SCH). By bolstering thyroid inflammation, anti-peroxidase antibody (TPO-Ab) causes autoimmune thyroiditis, which is one of the most common causes of SCH. Since the absence of thyroid cysts is associated with TPO-Ab (+) based on the indication of latent thyroid damage, we explored the potential mechanism underlying the association among TPO-Ab, SCH, hypertension, and thyroid cysts. A cross-sectional study of 1,483 Japanese aged 40–74 years was conducted. Thyroid cysts were defined as those having a maximum diameter of ≥ 2.0 mm, containing no solid component. TPO-Ab (+) was positively associated with SCH with hypertension (adjusted odds ratio [OR] and 95% confidence interval [CI], 2.62 [1.40, 4.89]) but not with SCH without hypertension (0.84 [0.37, 1.89]), respectively. Moreover, among participants without thyroid cysts, SCH was positively associated with hypertension (2.15 [1.23, 3.76]) but not among participants with thyroid cysts (0.58 [0.16, 2.16]), respectively. TPO-Ab was positively associated with SCH with hypertension, but not with SCH without hypertension. In addition, status of thyroid cysts might act as a determinant factor on the association between SCH and hypertension. These findings are efficient tools to clarify the background mechanism that underlies SCH.

## Introduction

Anti-thyroid peroxidase antibody (TPO-Ab) causes autoimmune thyroid disease [1] by bolstering thyroid inflammation [2]. In addition, high prevalence of TPO-Ab (+) is reported in subclinical hypothyroidism (SCH) [3,4]. Since both low-grade inflammations and hypothyroidism are known factors that are associated with hypertension [5,6], status of hypertension among SCH could act as a determinant factor of the association between TPO-Ab and SCH. Therefore, TPO-Ab (+) could be positively associated with SCH with hypertension but not with SCH without hypertension.

In addition, high concentration of thyroglobulin in the fluid from thyroid cysts was reported by a previous study [7]. Since thyroglobulin plays an important role in synthesizing thyroid hormone [8], thyroid cysts could possess beneficial effects on thyroid hormone synthesized by pooling thyroglobulin. Our previous study also reported a positive association between TPO-Ab titer and absence of thyroid cysts [9]. Since TPO-Ab injures thyroid, the absence of thyroid cysts might indicate latent thyroid damage. Moreover, since hypothyroidism is known to be associated with hypertension [6], this study speculated that SCH with absence of thyroid cysts could be associated with hypertension by indicating latent thyroid damage.

Furthermore, if the presence of thyroid cysts has a beneficial influence on thyroid hormone synthesis, thyroid cysts could be positively associated with isolated systolic hypertension because hyperthyroidism is known to be a common cause of isolated systolic hypertension [10].

Clarifying those associations by using multi-faceted analysis in a simple population is an efficient tool to clarify the background mechanism that underlies the association between TPO-Ab and SCH among eu-thyroid population.

We conducted a cross sectional study of 1483 eu-thyroid Japanese aged 40–74 years who participated in annual health check-up in 2014.

## Methods

### Study population

The methods that relate to the present risk survey including thyroid function have been described elsewhere [9].

We ensured that participants understood the objective of the study and informed consent was obtained. This study was approved by the Ethics Committee of Nagasaki, University Graduate School of Biomedical Sciences (Project registration number: 14051404).

In 2014, the total number of residents aged 40–74 years in Saza Town (estimated by the National Institute of Population and Social Security Research in March 2013) was 6,207 [11]. The study population comprised 1,883 Japanese individuals between the ages of 40 and 74 years from Saza town in the western part of Japan who underwent an annual medical checkup in 2014, as recommended by the Japanese government. The follow-up rate for this annual health check-up is high; more than 80% of individuals who underwent medical health check-up in 2014 also underwent medical health checkup in 2015. Participants without thyroid function data such as thyroid-stimulating hormone (TSH), free triiodothyronine (free T3), and free thyroxine (free T4) (n = 20), and participants with an abnormal free T3 (normal range: 2.1–4.1 pg/mL) and free T4 (normal range: 1.0–1.7 ng/dL) ranges were excluded (n = 78). Additionally, participants without TPO-Ab data (n = 302) were excluded.

The remaining 1,483 participants, with a mean age of 60.9 years (standard deviation [SD], 9.0; range 40–74), were enrolled in the study.

### Data collection and laboratory measurements

Trained medical staff conducted interviews to obtain clinical characteristic information such as that on past history of thyroid disease, medications taken for thyroid disease, and hypertension. Using a blood pressure measuring device (HEM-907; Omron, Kyoto, Japan), systolic blood pressure (SBP) and diastolic blood pressure (DBP) were recorded at and after more than 5 minutes rest.

Hypertension was defined as SBP ≥140 mmHg and /or DBP ≥90 mmHg and/or taking anti-hypertensive medication. Isolated systolic hypertension was defined as SBP ≥140 mmHg and DBP <90 mmHg. Isolated diastolic hypertension was defined as SBP <140 mmHg and DBP ≥ 90 mmHg.

A fasting blood sample was collected in the morning. TSH, free T3, and free T4, were measured by chemiluminescent Immunoassay (CLIA) and TPO-Ab was measured by electrochemiluminesnce immunoassay (ECLIA) at the LSI Medience Corporation (Tokyo, Japan). Normal range of free T3 (2.1–4.1 pg/mL), free T4 (1.0–1.7 ng/dL) TSH (0.39–4.01 μIU/mL) and TPO-Ab (<16 IU/mL) by using these methods were shown elsewhere [12]. Therefore, individuals with TSH >4.01 μIU/mL among present study population were diagnosed as having SCH. A positive value of TPO-Ab (+) was defined as at and above 16 IU/mL.

Thyroid cysts were detected by experienced technicians using a LOGIQ Book XP with a 10-MHz transducer (GE Healthcare, Milwaukee, WI, USA). A thyroid cyst (maximum diameter ≥2.0 mm) without a solid component was defined as a thyroid cyst for this study. The reproducibility of thyroid cyst detection based on ultrasonographic photo data of the study population (n = 20) was satisfactory. The respective intra-observer variations for detecting thyroid cysts, which were assessed by two examiners, were simple correlation coefficients (r) = 0.90 (p < 0.01) and r = 0.90 (p < 0.001), and the inter-observer variation was r = 0.70 (p < 0.001).

### Statistical analysis

TPO-Ab status-specific characteristics of the study population are expressed as mean ±SD except for TSH and prevalence data. Since TSH showed a skewed distribution, TSH was expressed as median [the first quartile, the third quartile], followed by logarithmic transformation. Significant differences by status of TPO-Ab were evaluated using analysis of variance (ANOVA). Hypertension specific characteristics of thyroid function in SCH were also expressed using ANOVA.

Logistic regression models were used to calculate odds ratios (ORs) and 95% confidence intervals (CIs) to determine the association between TPO-Ab and SCH and SCH subtypes categorized by hypertension status.

Logistic regression models were used to evaluate the association between SCH and hypertension by status of thyroid cysts.

In addition, we also evaluated the association between the status of thyroid cysts and hypertension (isolated systolic hypertension and isolated diastolic hypertension) among participants not taking anti-hypertensive medication, using logistic regression models.

Three adjustment models were used. The first model was adjusted only for sex-and age (model 1); the second model (model 2) further included the potential confounding factors that were directly associated with thyroid function, namely free T3 (pg/mL). As the medication for thyroid disease might act as a confounding factor in the present study, we used a further adjusted model to account for thyroid disease medication (no/yes) (model 3).

All statistical analyses were performed with the SAS system for Windows (version 9.4: SAS Inc., Cary, NC, USA). Values of p<0.05 were regarded as statistically significant.

## Results

Among the study population, 298 were diagnosed as TPO-Ab (+) and 89 were diagnosed as having SCH.

### Characteristics of study population by status of anti-thyroid peroxidase antibody (TPO-Ab)

TPO-Ab (+) shows significantly higher TSH value and higher prevalence of SCH than TPO-Ab (-) with significantly lower prevalence of thyroid cysts (Table 1).

**Table 1:** Characteristics of the study population by status of anti-thyroid peroxidase antibody (TPO-Ab).

	TPO-Ab		P
	(-)	(+)	
No at risk	1185	298	
Men, %	37.3	31.5	0.065
Age, year	60.7 ± 9.1	61.7 ± 8.6	0.068
TSH, (0.39–4.01), μIU/mL	1.53 [1.09, 2.21]*^1^	1.79 [1.16, 2.77]*^1^	0.001*^2^
free T3, (2.1–4.1), pg/mL	3.2 ± 0.3	3.2 ± 0.3	0.402
free T4, (1.0–1.7), ng/dL	1.2 ± 0.2	1.3 ± 0.2	0.389
Medication for thyroid disease, %	0.5	5.7	<0.001
Past history of thyroid disease, %	1.1	4.4	<0.001
Hypertension, %	40.4	43.6	0.316
Systolic blood pressure, mmHg	125 ± 17	126 ± 17	0.104
Diastolic blood pressure, mmHg	73 ± 10	74 ± 10	0.541
Anti-hypertensive medication, %	30.7	33.2	0.405
Subclinical hypothyroidism (SCH), %	5.2	9.1	0.013
Thyroid cysts, %	35.2	21.8	<0.001

TSH: thyroid stimulating hormone. free T3; free triiodothyronine, free T4; free thyroxine, Values are mean ±standard deviation.

*1: Values are median [the first quartile, third quartile].

*2: Logarithmic transformation was used for evaluating p. Normal range of measurements are ().

### Association between hypertension status and thyroid function in SCH

Hypertension status specific characteristics of thyroid function in SCH are shown in Table 2. While similar values of free T3 and T4 were found in SCH with and without hypertension, significantly higher values of TSH were observed in patients with SCH with hypertension than in those with SCH without hypertension.

**Table 2:** Association between hypertension status and thyroid function in patients with subclinical hypothyroidism (SCH).

	Hypertension		p
	(-)	(+)	
No. at risk	43	46	
Men, %	34.9	41.3	0.579
Age, year	58.7 ± 9.7	66.4 ± 6.6	<0.001
TSH, (0.39–4.01), μIU/mL	4.80 [4.34, 5.96]*^1^	5.75 [4.61, 6.47]*^1^	0.018*^2^
Free T3, (2.1–4.1), pg/mL	3.1 ± 0.3	3.1 ± 0.3	0.567
Free T4, (1.0–1.7), ng/dL	1.2 ± 0.2	1.2 ± 0.1	0.223
Medication for thyroid disease, %	4.7	6.5	0.706
Past history of thyroid disease, %	0	6.5	0.090

TSH: thyroid stimulating hormone. free T3; free triiodothyronine, free T4; free thyroxine, Values are mean ±standard deviation.

*1: Values are median [the first quartile, third quartile].

*2: Logarithmic transformation was used for evaluating p. Normal range of measurements are ().

### Association between subclinical hypothyroidism (SCH) and anti-thyroid peroxidase antibody (TPO-Ab)

Table 3 shows the ORs and 95% CIs of SCH for TPO-Ab. TPO-Ab (+) is significantly positively associated with SCH especially for SCH with hypertension. Those associations were unchanged even after further adjustment for free T3 and medication use for thyroid disease.

**Table 3:** Odds ratios and 95% confidence intervals of subclinical hypothyroidism (SCH) with respect to anti-thyroid peroxidase antibody (TPO-Ab).

	TPO-Ab		p
	(-)	(+)	
Subclinical hypothyroidism (SCH)			
No. at risk	1185	298	
No. of case (%)	62 (5.2)	27 (9.1)	
Model 1	1	1.77 (1.11, 2.85)	0.018
Model 2	1	1.78 (1.11, 2.87)	0.017
Model 3	1	1.63 (1.00, 2.67)	0.049
Subclinical hypothyroidism (SCH) with hypertension			
No. of case (%)	27 (2.3)	19 (6.4)	
Model 1	1	2.78 (1.51, 5.10)	0.001
Model 2	1	2.81 (1.53, 5.18)	<0.001
Model 3	1	2.62 (1.40, 4.89)	0.003
Subclinical hypothyroidism (SCH) without hypertension			
No. of case (%)	35 (3.0)	8 (2.7)	
Model 1	1	0.94 (0.43, 2.04)	0.867
Model 2	1	0.93 (0.43, 2.05)	0.868
Model 3	1	0.84 (0.37, 1.89)	0.665

Model 1: adjusted for sex and age. Model 2: + free T3. Model 3: + medication use for thyroid disease.

### Association between hypertension and SCH by thyroid cysts

Table 4 shows thyroid cysts status-specific association between SCH and hypertension. For participants without thyroid cysts, SCH was significantly positively associated with hypertension but not for participants with thyroid cysts. An investigation into the effects of the associations between thyroid cysts and SCH on hypertension revealed significant interactions.

**Table 4:** Odds ratios and 95% confidence intervals of hypertension in patients with subclinical hypothyroidism (SCH) with respect to thyroid cysts.

	Thyroid cysts						Interaction
	(-)			(+)			
	Subclinical hypothyroidism (SCH)		p	Subclinical hypothyroidism (SCH)		p	
	(-)	(+)		(⁻)	(+)		
No. at risk	941	60		453	29		
No. of case (%)	344 (36.6)	34 (56.7)		219 (48.3)	12 (41.4)		
Model 1	1	2.13 (1.19, 3.81)	0.011	1	0.65 (0.29, 1.44)	0.282	0.015
Model 2	1	2.16 (1.21, 3.87)	0.010	1	0.65 (0.29, 1.46)	0.297	0.014
Model 3	1	2.15 (1.23, 3.76)	0.007	1	0.58 (0.16, 2.16)	0.414	0.014

Model 1: adjusted for sex and age. Model 2: + free T3. Model 3: + medication for thyroid disease.

### Characteristics and the association between thyroid cysts and hypertension limited to participants not taking anti-hypertensive medication

Thyroid cysts’ specific characteristics of the participants who were not taking anti-hypertensive medication are shown in Table 5. Compared with participants without thyroid cysts, those with thyroid cysts were significantly older and tended to be women. Table 5 also shows the association between thyroid cysts and hypertension (isolated systolic hypertension and isolated diastolic hypertension) and shows that thyroid cysts were significantly positively associated with isolated systolic hypertension but not with isolated diastolic hypertension.

**Table 5:** Odds ratios and 95% confidence intervals of hypertension for thyroid cysts among participants not taking anti-hypertensive medication.

	Thyroid cysts		p
	(-)	(+)	
No. at risk	709	311	
Men	35.4	24.8	<0.001
Age, year	57.8 ± 9.5	61.2 ± 8.3	<0.001
TSH, (0.39–4.01), μIU/mL	1.57 [1.11, 2.22]*^1^	1.61 [1.08, 2.34]*^1^	0.373
free T3, (2.1–4.1), pg/mL	3.2 ± 0.3	3.1 ± 0.3	0.050
free T4, (1.0–1.7), ng/dL	1.2 ± 0.2	1.2 ± 0.1	0.108
Medication for thyroid disease, %	2.0	0.3	0.044
Past history of thyroid disease, %	1.3	1.9	0.421
Isolated systolic hypertension			
No. of case (%)	45 (6.3)	40 (12.9)	
Model 1	1	1.78 (1.12, 2.83)	0.016
Model 2	1	1.78 (1.11, 2.83)	0.016
Model 3	1	1.78 (1.12, 2.85)	0.016
Isolated diastolic hypertension			
No. of case (%)	8 (1.1)	3 (1.0)	
Model 1	1	0.84 (0.22, 3.28)	0.803
Model 2	1	0.83 (0.21, 3.24)	0.792
Model 3	1	0.81 (0.21, 3.16)	0.762

TSH: thyroid stimulating hormone. free T3; free triiodothyronine, free T4; free thyroxine, Values are mean ±standard deviation.

*1: Values are median [the first quartile, third quartile].

*2: Logarithmic transformation was used for evaluating p. Normal range of measurements are (). Model 1: adjusted for sex and age. Model 2: + free T3. Model 3: + medication for thyroid disease.

## Discussion

The major finding of this present study is that TPO-Ab is significantly positively associated with SCH with hypertension but not with SCH without hypertension in the eu-thyroid population. Moreover, the status of thyroid cysts might act as a determining factor in the association between SCH and hypertension.

**Fig 1:**
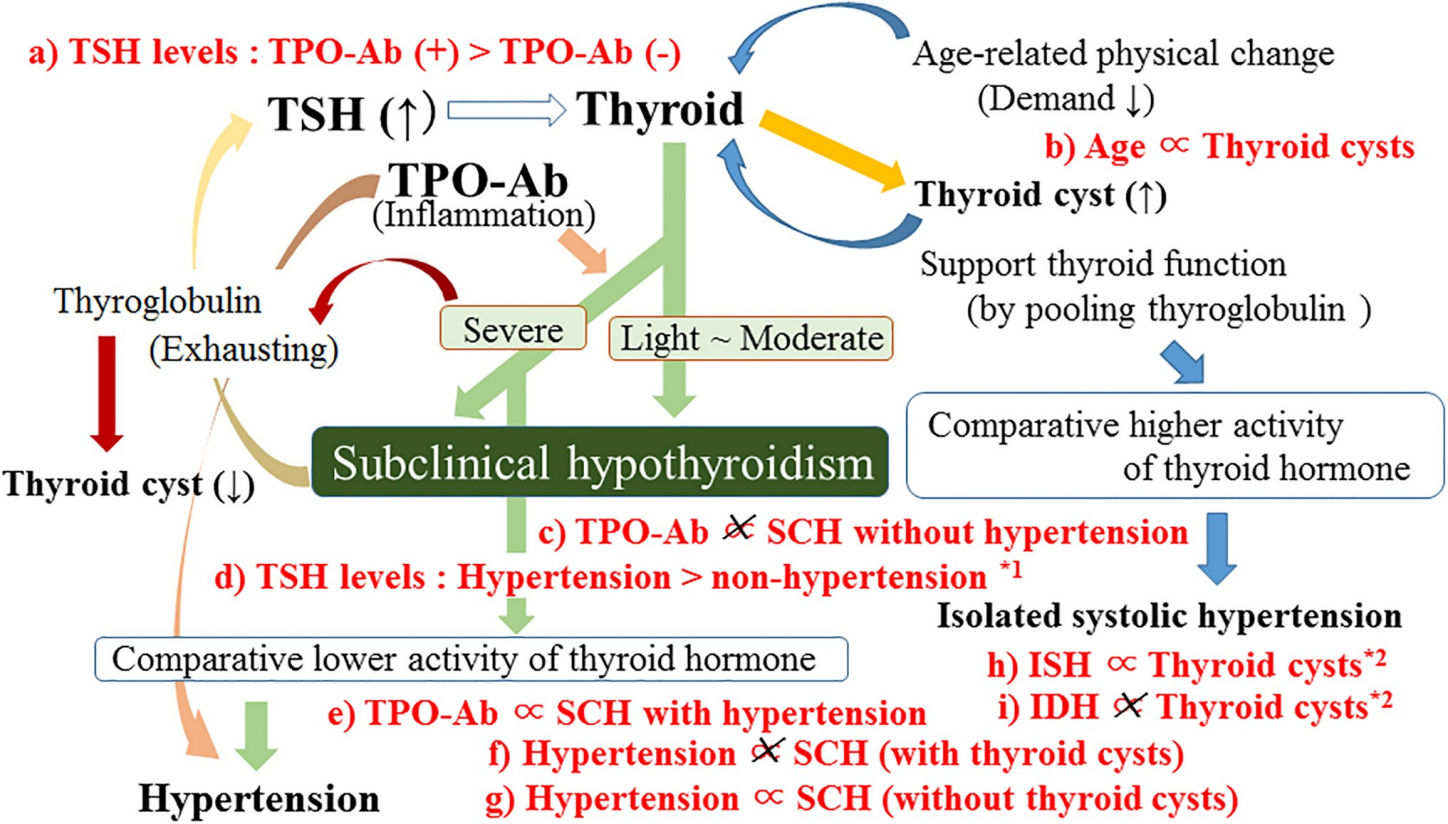
Potential mechanism underlying the present results. Associations shown in red (a ~i) were observed in the present study. TPO-Ab; anti-thyroid peroxidase antibody, TSH; thyroid stimulating hormone, SCH; Subclinical hypothyroidism, ISH: Isolated systolic hypertension. IDH: Isolated diastolic hypertension. *1: Observed among participants with SCH. *2: Observed among participants without taking anti-hypertensive medication.

The summary of the potential mechanism that underlies these present results was shown in Fig 1. TPO-Ab is positively associated with SCH with hypertension but not with SCH without hypertension. Thyroid cysts might support activity of thyroid function by pooling thyroglobulin that results in no significant association between SCH and hypertension.

Previous studies reported that the frequency of TPO-Ab (+) is high in SCH [3,4]. This is compatible with our present finding that TPO-Ab (+) is significantly positively associated with SCH (Table 3). In addition, we found further evidence that this significant positive association was limited to SCH with hypertension (Table 3, Fig 1c and 1e).

Hypothyroidism is a well-known cause of secondary hypertension [6]. A previous meta-analysis study reported significant association between SCH and increased blood pressure (both of systolic and diastolic) [13]. Therefore, even among SCH, those with hypertension might have a stronger biological effect of thyroid hormone deficiency than those without hypertension. In fact, in our present study, even the values of free T3 and free T4 show no significant difference between SCH participants with and without hypertension, SCH with hypertension shows significantly higher value of TSH than that of those without hypertension (Table 2, Fig 1d).

In this study, TPO-Ab (+) shows significantly higher values of TSH compared to TPO-Ab (-); whereas, essentially the same values were observed for free T3 and free T4 in both of them (TPO-Ab (+) and (-)) (Table 1, Fig 1a). As thyroid peroxidase, which has an important role in synthesizing thyroid hormone [14], is inhibited by TPO-Ab, latent thyroid damage induced by TPO-Ab might reduce the effectiveness of producing thyroid hormone, which elevates serum concentration of TSH.

In addition, a higher TPO-Ab titer is revealed to be associated with the absence of thyroid cysts among eu-thyroid participants possibly by indicating latent thyroid damage [9]. Since thyroglobulin is revealed to be rich in fluid from thyroid cysts [7], thyroglobulin plays important role in thyroid hormone synthesis [8], the presence of thyroid cysts might possess beneficial influence on thyroid hormone synthesized by pooling thyroglobulin. In other words, absence of thyroid cysts might have disadvantage in the production of thyroid hormone.

Hypothyroidism causes secondary hypertension [6] while absence of thyroid cysts might have disadvantage in production of thyroid hormone. In our present study, the positive association between SCH and hypertension is observed only for participants without thyroid cysts (Table 4, Fig 1f and 1g). This association also explained that subclinical hypothyroid with hypertension could possess more disadvantage in the production of thyroid hormone than that of subclinical hypothyroid without hypertension. Since this type of disadvantage could emphasize the influence of TPO-Ab, even positive association between TPO-Ab and SCH with hypertension had no significant association between TPO-Ab and SCH without hypertension was observed (Table 3, Fig 1c and 1e).

The prevalence of thyroid disturbances such as subclinical hypothyroidism increases with age [15]. Aging process might decrease the demand of thyroid hormone activity since decreased thyroid function may lead to extended longevity [16]. Therefore, thyroid cysts might be formed during aging partly by indicating comparatively excess portion of thyroid hormone. In the present study, we found participants with thyroid cysts significantly older than those participants without thyroid cysts; 60.1 ± 9.4 for participants without thyroid cysts (n = 1001) and 62.5 ± 7.9 for participants with thyroid cysts (n = 482) (p<0.001) (Table 5, Fig 1b). Under such a condition, the presence of thyroid cysts could have characteristics of higher thyroid hormone (hyperthyroidism) activity of even in eu-thyroid individuals.

Even both hypothyroidism and hyperthyroidism are associated with hypertension [6,10], diastolic hypertension is not common for hyperthyroidism. Thyroid hormone (T3) takes an important role in reducing the systemic vascular resistance [17,18] which lowers diastolic blood pressure while thyroid hormone (T3) enhance the force of myocardial contraction [19]. Therefore, hyperthyroidism was reported to be a common cause of isolated systolic hypertension [10]. Our additional analysis among participants without taking anti-hypertensive medication revealed that thyroid cysts are significantly positively associated with isolated systolic hypertension but not with isolated diastolic hypertension (Table 5, Fig 1h and 1i).

Participants with lower TSH and TPO-Ab (-) show high rate of reversion to eu-thyroidism while higher TSH were independently associated with progression to overt hypothyroidism among elderly participants with SCH (≥65 years) [20]. In this study, compared with non-hypertension, hypertension revealed significantly higher levels of TSH among participants with SCH. (Table 2, Fig 1d). Further investigations are necessary, present study provides efficient tool to risk estimation for progression of overt hypothyroidism.

To the best of our knowledge, this study is the first study that reveals that the status of thyroid cysts could act as a determinant factor on the association between SCH status and hypertension. In addition, this study is also the first study that revealed SCH subtyped by status of hypertension that might determine the association between TPO-Ab and SCH. Moreover, dissimilar to typical epidemiological study, our study used multi-faceted analysis that could support the potential mechanism underlying the major findings. Those are strength of our present study.

There is growing evidence suggesting that the treatment of SCH may not be beneficial, particularly in older patients [21]. However, aging patients may progress from SCH to overt hypothyroidism. Therefore, determining the timing to begin treatment for hypothyroidism is difficult in daily clinical practice [22]. Hypertension is a well-known strong health disturbance factor. As the present study demonstrates the potential mechanism that may underlie the association between SCH and hypertension, the present findings may provide efficient knowledge for daily clinical practice, although further investigation is necessary.

The potential limitations of this study warrant consideration. First, we evaluated the existence of a thyroid cyst on the parameters whether it was present or not. However, the number and size of a given cyst could also be an important factor and further investigation with this data is necessary. Due to the limited amount of blood samples, we could not evaluate the influence of anti-thyroglobulin-antibodies, which may act as a strong confounding factor. Further investigation with data of anti-thyroglobulin-antibody is necessary. Moreover, this was a cross-sectional study where a causal relationship could not be established. However, in this study we performed multi-faceted analyses that support the present results in a simple population model.

## Conclusion

In conclusion, TPO-Ab is significantly positively associated with SCH with hypertension but not with SCH without hypertension. In addition, status of thyroid cysts might act as a determinant factor on the association between SCH and hypertension. Those findings are efficient tools to clarify the background mechanism that underlying SCH.
